# Cardiac Implications of COVID-19 in Deceased and Recovered Patients: A Systematic Review

**DOI:** 10.1155/2022/9119930

**Published:** 2022-06-15

**Authors:** Rajaram Khanal, Shreeyash Raj Bhattarai, Kriti Basnet, Sangam Shah, Roman Dhital, Sanjit Kumar Sah, Sujan Poudel, Odalys Frontela, George Michel, Sima Marzban, Alfonso J. Rodriguez-Morales

**Affiliations:** ^1^Department of Cardiology, Manmohan Cardiothoracic Vascular and Transplant Center, Kathmandu, Nepal; ^2^Division of Research and Academic Affairs, Larkin Community Hospital, South Miami, FL, USA; ^3^Maharajgunj Medical Campus, Institute of Medicine, Tribhuvan University, Maharajgunj 44600, Nepal; ^4^Program Director, Internal Medicine Residency Program, Larkin Community Hospital- Palm Springs Campus, Hialeah, FL, USA; ^5^Program Director, Internal Medicine Residency Program, Larkin Community Hospital, South Miami, FL, USA; ^6^Grupo de Investigación Biomedicina, Faculty of Medicine, Fundación Universitaria Autónoma de Las Americas, Risaralda, Pereira, Colombia; ^7^Institución Universitaria Visión de Las Américas, Risaralda, Pereira, Colombia; ^8^School of Medicine, Universidad Privada Franz Tamayo, Cochabamba, Bolivia; ^9^Faculty of Health Sciences, Universidad Cientifica Del Sur, Lima, Peru

## Abstract

**Background:**

Patients infected with coronavirus disease 2019 (COVID-19) present with various clinical presentations with majority of them developing pulmonary complications. This study focuses on cardiac implications of COVID-19 which are less discussed and thus will help to address cardiac implications of COVID-19.

**Methods:**

PubMed, PubMed Central, and Google Scholar were screened for articles which mentioned cardiac implications of COVID-19. NHLBI Study Quality Assessment Tools for the observational cohort and cross-sectional studies was used for assessing the risk of bias of our studies.

**Results:**

All 14 studies selected were good and had score of ≥9 by NHLBI Study Quality Assessment Tools. Cardiac complications of COVID-19 are common. They are associated with significant mortality. Also, people infected with COVID-19 with premorbid conditions such as cardiovascular diseases and diabetes mellitus have poor prognosis as compared to those without premorbid conditions. Cardiac biomarkers such as highly sensitive troponin I, creatinine, and creatinine kinase-MB on admission are good prognostic markers.

**Conclusions:**

Cardiac complications such as heart failure, myocardial injury, and arrhythmias are common among patients infected with COVID-19. Elevated cardiac markers and patients with cardiac complications require utmost care and continuous cardiac monitoring.

## 1. Introduction

COVID-19 is an infectious disease caused by a newly identified novel enveloped *β*-coronavirus (SARS-CoV-2), first identified in the Wuhan, the capital city of Hubei province of China in December 2019 [1,2]. There are varied clinical presentations of COVID-19, ranging from asymptomatic state to severe disease, with some even resulting in the death of those affected. The most common symptoms that are presented are fever, cough, myalgia or fatigue, pneumonia, and complicated dyspnea with few presenting with other symptoms such as headache, diarrhea, running nose, and phlegm producing cough [3].

COVID-19 infection is associated with release of various inflammatory cytokines and chemokines which have a direct effect on the cardiovascular system resulting in various cardiac complications in addition to the common clinical presentation of respiratory failure [4, 5]. Moreover, a COVID-19 patient with preexisting CVD is more predisposed to the disease with a higher mortality rate [6, 7].

Most of the studies are conducted focusing on the pulmonary complications of COVID-19, with only a few mentioning the cardiac complications which are significantly responsible for mortality among COVID-19 patients. This study focuses on cardiac implications of COVID-19, both in terms of cardiac factors predisposing to the disease and cardiac complications of the disease, which will help to address the predisposing factors of COVID-19 and cardiac complications of the disease.

## 2. Methods

### 2.1. Protocol and Registration

**Figure 1:**
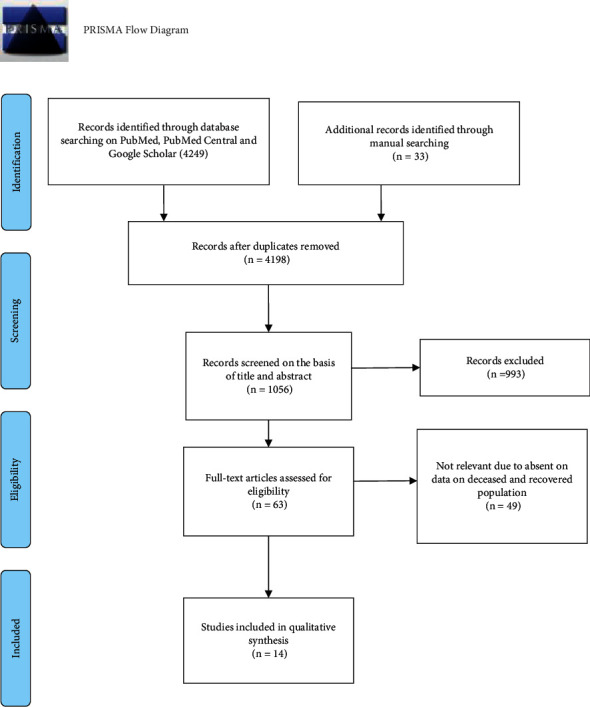
PRISMA flow diagram with flow of information during the systematic review.

We followed the Preferred Reporting Items for Systematic Reviews and Meta-Analyses (PRISMA) statement and checklist (10.6084/m9.figshare.14369600) for this systematic review [8]. (CRD42021288357) Article search and selection are detailed in the PRISMA diagram (Figure 1).

### 2.2. Eligibility Criteria

Articles published for peer reviews from January 1, 2020, to October 30, 2020, were included in the study. We included retrospective, prospective, and case series articles published in PubMed, PubMed Central (PMC), and Google Scholar. Case reports, letters to the editors, and editorials were excluded. Articles published in languages other than English were excluded. Only the articles including cardiovascular implications due to SARS-CoV-2 in recovered and deceased patients were included for the final study.

### 2.3. Search Strategy

Authors (SRB, KB, RD, SP, and SS) searched relevant articles indexed in PubMed, PubMed Central (PMC), and Google Scholar. Boolean operator “AND” was used for two separate key phrases. Authors reviewed the articles for repetition. For COVID-19, we used “COVID-19,” “SARS-CoV-2,” “coronavirus,” “nCoV-2019,” and “Novel Coronavirus 2019.” For cardiac implications we used “heart,” “cardiac,” “cardiovascular,” “acute myocardial injury,” “arrhythmia,” “heart failure,” and “myocardial infarction”. Final selection of the article was carried out by authors (SRB, KB, SP, RD, and SS) and verification was conducted by author (RRK). Further review of systematic reviews and meta-analyses revealed other relevant articles.

### 2.4. Data Extraction

First, the articles were screened by title and abstract, study design, and study site. Authors (SRB, KB, SP, RD, and SS) used Google Sheets for listing the articles by title, study design, and study site. Authors rechecked the spreadsheet to remove any duplicates. The articles were then reviewed for inclusion and exclusion criteria. Full-text review of the articles meeting the exclusion and inclusion criteria was conducted. A spreadsheet of articles for the final review was made in Google Sheets including title, author, journal of publication, country of conduct of research, study design, sample size, lab parameters on admission, signs and symptoms on admission, baseline characteristics of the sample, and cardiac complications which were verified by the author (RRK).

### 2.5. Inclusion Criteria

All studies that included patients diagnosed with COVID-19 by the Reverse Transcriptase-Polymerase Chain Reaction (RT-PCR) technique were included in the study. Also, only those articles from 1 January 2020 to 30 October 2020 which compared about the cardiac implications of COVID-19 between survivors and nonsurvivors were included in our study.

### 2.6. Exclusion Criteria

All articles beyond 30th October and those which did not compare between those patients who recovered from the disease from those who were deceased were excluded from our study due to limitations of study duration. Review articles and those articles which were not available in English were also excluded.

### 2.7. Assessment of Risk of Bias

We used the NHLBI Study Quality Assessment Tools for the observational cohort and cross-sectional studies (https://www.nhlbi.nih.gov/health-topics/study-quality-assessment-tools) for assessing the risk of bias of our studies. Each of 14 articles were evaluated independently by two authors (SRB and KB), and one author (RRK) assessed the decisions made.

### 2.8. Data Synthesis

All identified studies were included in the narrative summary with summary tables for characteristics. In addition, data were summarized using descriptive statistics. We used means for continuous variables and frequencies and percentages for dichotomous variables.

## 3. Results

### 3.1. Study Selection

The literature search resulted in 4282 studies from PubMed, PubMed Central (PMC), and Google scholar. After the complete screening process of titles, abstracts, and full texts, 4268 articles did not meet the eligibility criteria, and the 14 articles that met eligibility criteria were included in the review. The detailed description for the study selection is as shown in the PRISMA flow diagram (Figure 1).

All the studies that we included were the retrospective cohort study. Most of the studies were from China, but Amit et al., Alamdari et al., and Stefano Ghio et al. are from Israel, Iran, and Italy, respectively [9–11]. The sample size and outcomes (i.e., survivors and nonsurvivors) of our selected studies are listed in Table 1.

### 3.2. Comorbidities

#### 3.2.1. Hypertension

In a study by Wang et al., out of 26 patients with hypertension, there were only 16 survivors (*p*=0.001) [12]. Similarly, in a study by Zhou et al., only 32 out of 58 patients with hypertension survived (*p*=0.0008) [14]. A study in Israel by Amit et al. of 85 patients with hypertension, 23 patients were found to be dead, and 62 survived (*p*=<0.0001) [9]. Similar findings were found in studies by Shi et al., Fang-fang Chen et al., and Yukun et al. where number of patients who survived were more than dead, and *p* value was also significant, but in studies by Alamdari et al. and Si et al., *p* value was not significant [14–17,22]. However, Wang et al., Deng et al., Chen et al., and Xie et al. reported more deaths among patients who had hypertension, and data were statistically significant [18–21].

#### 3.2.2. Cardiovascular Disease (CVD)

More deaths were found in patients with CVD, and *p* value was statistically significant in studies by Wang et al., Yang et al., Zhou et al., Chen et al., Deng et al., Wang et al., Amit et al., and Yukun et al., but Shi et al., Fang-fang Chen et al., and Stefano Ghio et al. reported more survivors among patients with CVD with a significant *p* value. *p* value among survivors and deceased was not significant in the study conducted by Alamdari et al. [9–14,16,17,19–21].

#### 3.2.3. Diabetes Mellitus (DM)

More deaths were found among the patients with DM than those without it in studies by Si et al. (*p*=0.7) and Deng et al. (*p*=0.066), but *p* value was not statistically significant [15,20]. Xie et al., Chen et al., Wang et al., Amit et al., and Yukun et al. reported a statistically significant number of deaths than those who survived [9,18,19,21,22]. Studies by Wang et al., Zhou et al., Shi et al., Alamdari et al., and Stefano Ghio et al. reported more survivors [10–12,14,16].

#### 3.2.4. Chronic Obstructive Pulmonary Disease (COPD)

The studies by Si et al., Xie et al., Wang et al., and Amit et al. showed more deaths among patients with COPD, but *p*value was not significant; however, studies by Deng et al., Shi et al., Fang-fang Chen et al., Alamdari et al., and Stefano Ghio et al. reported more statistically significant survivors than deaths among patients with COPD [9–11,15–18,20,21].

#### 3.2.5. Chronic Kidney Disease (CKD)

In studies by Zhou et al., Si et al., Xie et al., and Chen et al., the number of nonsurvivors was found to be higher among patients with CKD, but the *p*value was insignificant; however, the studies by Amit et al., Stefano Ghio et al., and Wang et al. showed statistically significant deaths [9,11,14,15,18,19,21]. Among the studies where the number of survivors was higher, Wang et al., Yang et al., and Fang-fang Chen et al. showed an insignificant *p* value; whereas the *p* value reported by Shi et al. and Alamdari et al. was significant [10,12,13,16,17].

#### 3.2.6. Cerebrovascular Accident (CVA)

Study by Si et al., Xie et al., Chen et al., and Deng et al. showed statistically significant deaths among patients who had CVA coexisting with COVID-19 [15,18–20].

The table comparing the comorbidities between the survivors and nonsurvivors is listed in Table 2.

### 3.3. Symptoms

Fever, cough, dyspnoea, myalgia, headache, diarrhoea, and fatigue were the most common symptoms that appeared in the patients with COVID-19. Most of the studies showed more number of survivors than nonsurvivors with these symptoms [12–14,16,17]. There was no association of fever, myalgia, diarrhea, and headache with cardiovascular factors [10,12,20]. There was association of dyspnoea with cardiovascular factors [11,12,18,19]. The details of different symptoms between survivors and nonsurvivors are as shown in Table 3.

### 3.4. Laboratory Parameters and Cardiovascular Complications of COVID-19

In the study conducted by Wang et al., 22 out of total 107 sample population (patients) developed shock of which 19 did not survive [12]. Only 4 out of 12 patients who developed acute myocardial injury (hs-TnI >26.2 pg/ml) survived. His study also showed that levels of creatine phosphokinase-MB (CPK-MB) (*p*=0.008), troponin I (*p*=0.001), D-dimer (*p*=0.003), and creatinine (*p* < 0.001) were significantly higher in nonsurvivors than in survivors [12]. Univariate analysis by Wang et al. also concluded elevated lactate dehydrogenase and creatinine at admission to be an independent risk factor for mortality [12].

Similarly, Zhou et al. found a statistically significant number of nonsurvivors among patients developing shock, heart failure, and acute myocardial injury (*p* < 0.0001) [14]. All of 38 patients who developed shock died, 32 out of 33 patients who developed acute myocardial injury (highly sensitive- troponin I (hs-TnI) >28 pg/ml), and 52% (*n* = 28/44) patients who developed heart failure died. Zhou et al. also found that creatinine kinase (CK), troponin I, and D-dimer were significantly higher in nonsurvivors (*p* < 0.001) [14].

Of 671 study population of Shi et al., 62 did not survive. Out of these, 20 (30.6%) died of acute myocardial injury, 4 (6.5%) died of shock, and 12 (19.4%) died of heart failure [16]. It was also found that levels of CPK-MB (*p* < 0.001), procalcitonin (*p* < 0.001), troponin I (*p* < 0.001), hs-CRP (*p* < 0.001), probrain natriuretic peptide (pro-BNP) (*p* < 0.001), and creatinine (*p* < 0.001) were significantly high among nonsurvivors.

Chen et al. considered troponin I above 15.6 pg/ml to be a sign of myocardial injury [19]. He has also concluded shock, acute myocardial injury, and heart failure as cardiac complications associated with a greater number of deaths. He has also found higher values of CK, procalcitonin, troponin I, pro-BNP, creatinine, D-dimer, and highly sensitive-C reactive protein (hs-CRP) among the nonsurvivors.

Deng et al. found that all 13 of his patients who developed shock died (*p* < 0.001), and only 1 of the 66 patients who developed acute myocardial injury survived suggesting a statistically significant number of deaths due to acute myocardial injury in COVID-19-infected patients (*p* < 0.001) [20]. His study also found higher average values of hs-CRP among nonsurvivors (109.25 mg/l; 35.00–170.28) than survivors (3.22 mg/l; 1.04–21.80).

Another study by Wang et al. also found significant cardiac complications like shock (51/56) and acute myocardial injury (67/72) among nonsurvivors (*p* < 0.001) [21]. However, CPK-MB was not raised among the nonsurvivors in comparison with CK, procalcitonin, and D-dimer which were significantly higher among nonsurvivors of the study group (*p* < 0.001).

Similarly, Amit et al. also found higher D-dimer and creatinine levels among the nonsurvivors (*p* < 0.001). 20 out of 29 patients (*p* < 0.001) who developed shock did not survive [9]. 8 people of his study group developed acute myocardial injury of which only 3 survived (*p*=0.694) [9]. 9 people who developed heart failure in his sample size did not survive (*p*=0.079) [9].

Of 469 sample population of Alamdari et al., 63 lost their lives [10]. A total of 53 patients during the time frame developed some form of cardiac arrhythmia of which 30 did not survive (*p* < 0.001) [10]. 3.39 ± 2.94 mg/dl was the average value of D-dimer among the nonsurvivors which is significantly higher than the people who survived (*p* < 0.001) [10]. Also, the lab values of creatinine were higher among the group of people who did not survive (*p* < 0.001) [10].

In hospitals, mortality was higher in patients with raised TnI (31 ng/L; 15–80) as compared to the patients who survived (11 ng/L; 5–25) (*p* < 0.001) in the sample population of Stefano Ghio et al. [11]. Also, lab values of creatinine were higher among the nonsurvivors (*p* < 0.001) [11]. 51 of the 82 patients who developed heart failure survived in his study population (*p*=0.25) [11]. However, a significant number of people (17 out of 29) who developed arrhythmia died (*p*=0.002) [11].

Fang-fang Chen et al. also found that higher levels of CPK-MB, troponin I, hs-CRP, pro-BNP, and creatinine were significantly high among nonsurvivors (*p* < 0.001) [17]. Si et al. has included only the population with elevated troponin I in his study [15]. He also concluded higher association of troponin I values with increasing chances of fatality (*p* < 0.001). Of the 170 study populations of Si et al., 44 developed arrhythmias of which 6 died. 20% (*n* = 147) of 733 of the study population of Xie et al. developed shock [15,18]. Also, 59.2% (357) developed acute myocardial injury (hs- TnI >26 pg/ml). Troponin I and D-dimer were also raised among the people who did not survive (*p* < 0.001) in the study population of Yukun et al. [22]. The comparison of lab parameters and cardiovascular complications of COVID-19 of the studies between the survivors and nonsurvivors are given in Tables 4 and 5, respectively.

### 3.5. Assessment of Risk of Bias in Selected Studies

The NHLBI scoring assessing the risk of bias in selected studies is attached in supplementary file 1. All the studies are good with 12 out of 14 studies which can score ≥10. Only two articles had scores less than that with 9/13 in that of Shi et al. and 9/14 in Stefano Ghio et al. [11,16]. The average score of the included study was of 10.07 (good quality). Although the data were of good quality, we did not perform meta-analysis due to lack of homogenous data among the included studies. Same parameters were reported as different unit values.

## 4. Discussion

Patients of relatively older age are more susceptible to SARS COVID-19 infection, and this contributed to the poor prognosis among the patients [10,17]. In our study, the average age of morbid patients was found to be 71.18 ± 19.43 compared to 56.88 ± 21.6 in survivors implying that as age increases, the probability of morbidity increases in patients infected with COVID-19 [9–22]. Age was predicted to be an independent risk factor for mortality among patients with COVID-19 from multivariate analysis, and similar findings were observed in patients who had SARS and MERS [12,23,24]. Male sex was found to be more affected with COVID-19 than females and had higher mortality [10,25]. A probable reason for this could be steroidal hormones, sex chromosomal, or due to specific innate immunity among females. Sex was also found to be an independent risk factor for mortality by multivariate analysis by Wang et al. [12]. But there was no distinction between discharged and deceased patients in a study by Yukun et al. suggesting gender is not a risk factor for death [22].

COVID-19 is a viral infection due to pathogens residing in the nasopharynx [10]. In patients with COVID-19 symptoms such as fever, myalgia, cough, and fatigue were present; there was no significant difference in symptom presentation between survivors and nonsurvivors. However, dyspnoea was more common in nonsurvivors [12,16,17]. Indicators of hypoxemia are used to evaluate the severity of patients with COVID-19 [26].

The most common comorbidity among patients with COVID-19 was hypertension [12,14,15,18]. This could be due to use of ACE inhibitors, as they indirectly increase cellular ACE2 receptors which could be a receptor for COVID-19 [20]. Since ACE receptors are also present in kidneys and lungs so, patients having underlying kidney and lung disease could also be affected. Patients with comorbidities such as lung diseases, heart diseases, and malignancy were found to be an independent risk factor to predict mortality among critically ill patients of COVID-19 [11,27]. Multivariate regression analysis showed that there was no relation in the independent predictor for mortality and the comorbidities among patients with COVID-19. As diabetes increases the risk of infection and delays the recovery, it is one of the major comorbidities among the patients and is associated with the mortality with COVID-19, and similar findings were observed in patients who had severe acute respiratory syndrome (SARS) and Middle East respiratory syndrome (MERS) [28,29]. But in a study by Deng et al. [20], they found no association between the deceased and survivors in patients with diabetes.

In our study, the most common presentation to the hospital was with fever followed by cough. All articles except by Amit et al. and Wang et al. found that cardiac biomarkers such as CPK-MB, CK, procalcitonin, troponin I, D-dimer, hs-CRP, pro-BNP, and creatinine were high among nonsurvivors when measured (*p* < 0.05) [9,21]. Thus, these biomarkers may have a prognostic use in COVID-19 infections. Also, patients with elevated cardiac enzymes in COVID-19 infections can be categorized as high-risk patients.

In addition to reverse transcriptase polymerase chain reaction (RT-PCR), a convolutional neural network (CNN) with direct usage of CT scan images has been identified for patient diagnosis [30]. The CNN architecture has a greater accuracy (93.2%) and sensitivity (96.1%). With great performance, the provided CNN architecture can be used to diagnose COVID-19 patients as the RT-PCR method is both costly and time-consuming. Simvastatin has a good effect on COVID-19 severity in persons who take it before getting infected with the virus, according to a study by Davoudi et al. [31]. Furthermore, the decision tree method was discovered to be a useful tool for predicting the severity of patients based on clinical symptoms. The HSSAGA model for designating and scheduling of nurses for taking care of COVID-19 patients using a novel method of hybrid salp swarm algorithm and genetic algorithm has been developed for solving nurses' scheduling and designation [32].

Our study had many limitations. Most of the studies that are included in this review are from China, and the study from other countries are lacking. The studies that we included were only retrospective, due to which all the data required are not available; observational studies are lacking. The postdischarge follow-up duration was short while follow-up time in the hospital was long compared with the course of the disease in most of the studies; hence, the mortality rate and duration of hospital stay may have been varied. The sample size of most of the studies included was small. The authors did not perform meta-analysis for the topic because of lack of homogeneity of the study variables as some articles expressed those in percentage whereas others in numbers. Also, not all variables of the articles were comparable to each other with many variable parameters missing in different articles.

## 5. Conclusion

Shock, acute cardiac injury, arrhythmias, and heart failure were common cardiac complications with COVID-19. Patients with these complications were found to have a higher statistically significant morbidity. Thus, patients with these complications have poor prognosis and must be monitored carefully with importance, and cardiac care must be given to patients with these complications and elevated cardiac markers.

## Figures and Tables

**Table 1:** Characteristics of included study.

Author	Country of study	Study design	Sample size	Outcomes	
				Survivors	Nonsurvivors
Wang et al. [12]	China	Retrospective	107	88	19
Yang et al. [13]	China	Retrospective	52	20	32
Zhou et al. [14]	China	Retrospective	191	137	54
Si et al. [15]	China	Retrospective	170	49	121
Shi et al. [16]	China	Retrospective	671	609	62
Fang-fang Chen et al. [17]	China	Retrospective	681	577	104
Xie et al. [18]	China	Retrospective	733	339	394
Chen et al. [19]	China	Retrospective	274	161	113
Deng et al. [20]	China	Retrospective	225	116	109
Wang et al. [21]	China	Retrospective	293	177	116
Amit et al. [9]	Israel	Retrospective	156	69	87
Alamdari et al. [10]	Iran	Retrospective	459	396	63
Stefano Ghio et al. [11]	Italy	Retrospective	405	281	124
Yukun et al. [22]	China	Retrospective	101	66	35

**Table 2:** Comorbidities of the patients at the time of presentation.

	Hypertension	CHD or CVD	Diabetes mellitus	COPD	CKD	CVA/stroke
Author	Total	Total	Total	Total	Total	Total
	Survivor	Survivor	Survivor	Survivor	Survivor	Survivor
	Nonsurvivor	Nonsurvivor	Nonsurvivor	Nonsurvivor	Nonsurvivor	Nonsurvivor
	*p* value	*p* value	*p* value	*p* value	*p* value	*p* value

Wang *et al.* [11]	26 (24.3)	13 (12.1)	11 (10.3)	3 (2.8)	3 (2.8)	6 (5.6)
	16 (18.2)	6 (6.8)	6 (6.8)	2 (2.3)	2 (2.3)	3 (3.4)
	10 (52.6)	7 (36.8)	5 (26.3)	1 (5.3)	1 (5.3)	3 (15.8)
	0.001	0.002	0.024	0.447	0.447	0.068

Yang *et al.* [13]	NA	5 (10)	9 (17)	4 (8)	NA	NA
		2 (10)	2 (10)	2 (10)		7 (13.5)
		3 (9)	3 (9)	2 (6)		7 (22)
		NA	NA	NA		NA

Zhou *et al.* [14]	58 (30)	15 (8)	36 (19)	6 (3)	2 (1)	NA
	32 (23)	2 (1)	19 (14)	2 (1)	0	
	26 (48)	13 (24)	17 (31)	4 (7)	2 (4)	
	0.0008	<0.0001	0.0051	0.047	0·024	

Si *et al.* [15]	NA	NA	NA	NA	NA	NA
	30 (65.2)	9 (18.4)	12 (24.5)	1 (2)	4 (8.2)	1 (2)
	65	21 (17.4)	25 (20.7)	10 (8.3)	5 (4.1)	5 (4.1)
	1	1	0.7	0.2	0.3	0.7

Shi *et al.* [16]	199 (29.7)	60 (8.9)	97 (14.5)	23 (3.4)	28 (4.2)	22 (3.3)
	162 (26.6)	39 (6.4)	80 (13.1)	21 (3.4)	16 (2.6)	14 (2.3)
	37 (59.7)	21 (33.9)	17 (27.4)	2 (3.2)	12 (19.4)	8 (12.9)
	<0.001	<0.001	0.004	1	<0.001	<0.001

Fang-fang Chen *et al.* [17]	293 (43)	80 (11.7)	114 (16.7)	15 (2.2)	27 (4)	33 (4.8)
	227 (39.3)	55 (9.5)	96 (16.6)	13 (2.3)	20 (3.5)	20 (3.5)
	66 (63.5)	25 (24)	18 (17.3)	2 (1.9)	7 (6.7)	13 (12.5)
	0	0	0.866	1	0.194	0

Xie *et al.* [18]	308 (42)	93 (12.7)	138 (18.8)	37 (5)	13 (1.8)	34 (4.6)
	122 (36)	38 (11.2)	60 (17.8)	13 (3.8)	5 (1.5)	14 (4.1)
	186 (47.2)	55 (14)	78 (19.8)	24 (6.1)	8 (2)	20 (5.1)
	0.002	0.265	0.469	0.164	0.57	0.544

Chen *et al*. [19]	93 (34)	23 (8)	47 (17)	18 (7)	5 (1)	4 (1)
	39 (24)	7 (4)	23 (14)	7 (4)	1 (1)	0
	54 (48)	16 (14)	24 (21)	11 (10)	4 (4)	4 (4)
	NA	NA	NA	NA	NA	NA

Deng *et al.* [20]	NA	NA	NA	NA	NA	NA
	18 (15.5)	4 (3.4)	9 (7.8)	3 (2.6)		
	40 (36.7)	13 (11.9)	17 (15.6)	22 (20.2)		
	<0.001	0.031	0.066	<0.001		

Wang *et al.* [21]	92 (31.4%)	21 (7.2%)	37 (12.6%)	11 (3.8%)	11 (3.8%)	22 (7.5%)
	26 (14.7%)	7 (4.0%)	14 (7.9%)	4 (2.3%)	2 (1.1%)	3 (1.7%)
	66 (56.9%)	14 (12.1%)	23 (19.8%)	7 (6.0%)	9 (7.8%)	19 (16.3%)
	<0.001	0.009	0.003	0.097	0.004	<0.001

Amit *et al.* [9]	85 (54.5)	33 (21.2)	62 (39.7)	13 (8.3)	24 (15.4)	NA
	23	9	20	3	4	
	62	24	42	10	20	
	<0.00001	0.03	0.01	0.1	0.003	

Alamdari *et al.* [10]	214 (46.6%)	185 (40.3%)	119 (25.19%)	32 (28.8%)	99 (21.6%)	NA
	188 (47.5%)	156 (39.4%)	95 (24.0%)	109 (27.5%)	76 (19.2%)	
	26 (41.3%)	29 (46%)	24 (38.1%)	23 (36.5%)	23 (36.5%)	
	0.359	0.318	0.018	0.143	0.002	

Stefano Ghio *et al.* [11]	NA	NA	NA	NA	NA	NA
		166 (60.4%)	43 (15.9%)	39 (14.4%)	16 (5.9%)	
		102 (84.3%)	36 (29.8%)	19 (15.7%)	22 (18.2%)	
		<0.001	0.002	0.74	<0.001	

Yukun *et al.* [22]	38 (37.6%)	21 (20.8%)	18 (17.8%)	16 (15.8%)	NA	NA
	20 (30.3%)	10 (15.2%)	6 (9.1%)	9 (25.7%)		
	18 (51.4%)	11 (31.4%)	12 (34.3%)	7 (10.6%)		
	0.037	0.055	0.002	0.048		

NA= not applicable.

**Table 3:** Symptoms of the patients at the time of presentation.

	Fever	Cough	Dyspnoea	Myalgia	Headache	Diarrhoea
Author	Total	Total	Total	Total	Total	Total
	Survivor	Survivor	Survivor	Survivor	Survivor	Survivor
	Nonsurvivor	Nonsurvivor	Nonsurvivor	Nonsurvivor	Nonsurvivor	Nonsurvivor
	*p* value	*p* value	*p* value	*p* value	*p* value	*p* value

Wang *et al.* [12]	104 (97.2)	67 (62.6)	35 (32.7)	33 (30.8)	7 (6.5)	7 (6.5)
	85 (96.6)	56 (63.6)	20 (22.7)	28 (31.8)	7 (8.0)	3 (3.4)
	19 (100.0)	11 (57.9)	15 (78.9)	5 (26.3)	0 (0)	4 (21.1)
	1	0.639	<0.001^*∗*^	0.638	0.348	0.018

Yang *et al.* [13]	51 (98%)	40 (77%)	33 (63·5%)	6 (11·5%)	3(6%)	NA
	20 (100%)	15 (75%)	12 (60%)	2 (10%)	1 (5%)	
	31 (97%)	25 (78%)	21 (66%)	4 (12·5%)	2 (6%)	
	NA	NA	NA	NA	NA	

Zhou *et al.* [14]	180 (94%)	151 (79%)	NA	29 (15%)	NA	9 (5%)
	129 (94%)	112 (82%)		21 (15%)		7 (5%)
	51 (94%)	39 (72%)		8 (15%)		2 (4%)
	0·94	0·15		0.93		0·67

Si *et al.* [15]	NA	NA	NA	NA	NA	NA
	41 (83.7)	30 (61.2)	15(30.6)			12 (24.5)
	101 (71.1)	76 (62.8)	62 (51.2)			25 (20.7)
	1	0.9	0.02			0.7

Shi *et al.* [16]	NA	NA	NA	NA	NA	NA

Fang-fang Chen *et al.* [17]	584 (85.9%)	462 (67.8%)	123 (18.1%)	NA	NA	119 (17.5%)
	494 (85.8%)	397 (68.8%)	95 (16.5%)			104 (18.1%)
	90 (86.5%)	65 (62.5%)	28 (26.9%)			15 (14.4%)
	0.835	0.205	0.011			0.366

Xie *et al.* [18]	630 (85.9)	550 (75)	444 (60.7)	NA	NA	90 (12.3)
	287 (84.7)	254 (74.9)	163 (48.1)			44 (13)
	343 (87.1)	296(75.1)	281 (71.3)			46 (11.8)
	0.352	0.95	<0.001			0.592

Chen *et al*. [19]	249 (91)	185 (68)	120 (44)	60 (22)	31 (11)	77 (28)
	145 (90)	106 (66)	50 (31)	39 (24)	20 (12)	50 (31)
	104 (92)	79 (70)	70 (62)	21 (19)	11 (10)	27 (24)
	NA	NA	NA	NA	NA	NA

Deng *et al.* [20]	189	85	99	57	13	33
	94 (81.0)	38 (32.8)	22 (19.0)	27 (23.3)	7 (6.0)	14 (12.1)
	95 (87.2)	47 (43.1)	77 (70.6)	30 (27.5)	6 (5.5)	19 (17.4)
	0.211	0.109	<0.001	0.464	0.865	0.252

Wang *et al.* [21]	209 (71.3%)	150 (51.2%)	81 (27.6%)	17 (5.8%)	10 (3.4%)	19 (6.5%)
	119 (67.2%)	81 (45.8%)	33 (18.6%)	11 (6.2%)	7 (4.0%)	16 (9.0%)
	90 (77.6%)	69 (59.5%)	48 (41.4%)	6 (5.2%)	3 (2.6%)	3 (2.6%)
	0.055	0.022	<0.001	0.709	0.528	0.028

Amit *et al.* [9]	NA	NA	NA	NA	NA	NA

Alamdari *et al.* [10]	384 [83.7%]	251 [54.7%]	NA	284 [61.9%]	89 [19.4%]	125 [27.2%]
	326 [82.3%]	213 [53.8%]		247 [62.4%]	78 [19.7%]	105 [26.5%]
	58 [92.1%]	38 [60.3%]		37 [58.7%]	11 [17.5%]	20 [31.7%]
	0.052	0.344		0.58	0.677	0.386

Stefano Ghio *et al.* [11]	NA	NA	NA	NA	NA	NA
			171 (62.2%)			
			93 (75.6%)			
			0.009			

Yukun *et al.* [18]	96 (95%)	79 (78.2%)	17 (16.8%)	13 (12.9%)	NA	9 (8.9%)
	62 (93.9%)	53 (80.3%)	8 (12.1%)	8 (12.1%)		6 (9.1%)
	34 (97.1%)	26 (74.3%)	9 (25.7%)	5 (14.3%)		3 (8.6%)
	0.656	0.486	0.082	0.757		1

NA= not applicable.

**Table 4:** Laboratory parameters of the included patients among the studies.

	CPK MB (in ng/ml)	CK (in U/L)	Procalcitonin (in ng/ml)	Troponin I (ng/ml)	D-dimer (*µ*g/L)	Hs-CRP (mg/L)	Pro-BNP (pg/ml)	Creatinine (mg/dl)
Author	Total	Total	Total	Total	Total	Total	Total	Total
	Survivor	Survivor	Survivor	Survivor	Survivor	Survivor	Survivor	Survivor
	Nonsurvivor	Nonsurvivor	Nonsurvivor	Nonsurvivor	Nonsurvivor	Nonsurvivor	Nonsurvivor	Nonsurvivor
	*p* value	*p* value	*p* value	*p* value	*p* value	*p* value	*p* value	*p* value

Wang *et al.* [12]	14 (10–18)^*∗*^	90 (54–138)	NA	6 (5.6)^*∗∗*^	203 (121–358)^*∗∗∗*^	NA	NA	71 (60–86)^*∗∗∗∗*^
	13 (9–16)^*∗*^	86 (53–121)		1 (1.1)^*∗∗*^	191 (108–327)^*∗∗∗*^			68 (58–83)^*∗∗∗∗*^
	18 (13–44)^*∗*^	142 (87–325)		5 (26.3)^*∗∗*^	439 (202–1991)^*∗∗∗*^			87 (71–130)^*∗∗∗∗*^
	0.008	0.022		0.001	0.003			<0.001

Yang *et al.* [13]	NA	NA	NA	NA	NA	NA	NA	NA
								76.3 (27·4)^*∗∗∗∗*^
								80.7 (32·3)^*∗∗∗∗*^
								NA

Zhou *et al.* [14]	NA	21.5 (13·0–72·4)	0.1 (0·1–0·1)	4.1 (2·0–14·1)	0.8 (0·4–3·2)	NA	NA	NA
		18.0 (12·5–52·1)	0.1 (0·1–0·1)	3.0 (1·1–5·5)	0.6 (0·3–1·0)			
		39.0 (19·5–151·0)	0.1 (0·1–0·5)	22.2 (5·6–83·1)	5.2 (1·5–21·1)			
		0.001	<0.0001	<0.0001	<0.0001			

Si *et al.* [15]	NA	NA	NA	NA	NA	NA	NA	NA
				48.5 (30.8, 128.1)^*∗∗*^				
				393.8 (139.9, 1700)^*∗∗*^				
				< 0.001				

Shi *et al.* [16]	0.96 (0.63–1.82)	NA	0.06 (0.04–0.13)	0.006 (0.006–0.016)	NA	41 (12–81)	189 (67–494)	58 (48–70)^*∗∗∗∗*^
	0.8 (0.6–1.2)		0.05 (0.03–0.09)	0.006 (0.006–0.011)		30 (8–59)	132 (58–237)	55 (48–63)^*∗∗∗∗*^
	3.6 (2.4–6.9)		0.46 (0.14–1.58)	0.235 (0.042–1.996)		111 (64–191)	1819 (759–5164)	87 (59–160)^*∗∗∗∗*^
	<0.001		<0.001	<0.001		<0.001	<0.001	<0.001

Fang-fang Chen *et al*. [17]	1.09 (0.70–2.00)	NA	NA	0.00 (0.00–0.03)	NA	40.1 (7.4–82.3)	NA	61.0 (50.0–74.5)^*∗∗∗∗*^
	0.99 (0.66–1.51)			0.00 (0.00–0.01)		29.4 (5.0–68.6)		60.0 (50.0–72.0)^*∗∗∗∗*^
	3.27 (1.72–5.95)			0.15 (0.03–0.78)		101.0 (59.1–180.7)		73.0 (54.0–112.0)^*∗∗∗∗*^
	0			0		0		0

Xie *et al.* [18]	NA	NA	NA	NA	NA	NA	NA	NA

Chen *et al*. [19]	NA	109.0 (53.5–188.0)	0.09 (0.04–0.23)	8.7 (2.9–33.6)^*∗∗*^	1.1 (0.5–3.2)	53.4 (18.6–113.0)	267.0 (48.0–821.0)	76.0 (58.0–94.0)^*∗∗∗∗*^
		84.0 (50.8–140.3)	0.05 (0.03–0.08)	3.3 (1.9–7.0)^*∗∗*^	0.6 (0.3–1.3)	26.2 (8.7–55.8)	72.0 (20.0–185.0)	66.0 (54.0–84.0)^*∗∗∗∗*^
		189.0 (94.5–374.5)	0.33 (0.14–0.65)	40.8 (14.7–157.8)^*∗∗*^	4.6 (1.3–21.0)	113.0 (69.1–168.4)	800.0 (389.8–1817.5)	88.0 (66.0–114.0)^*∗∗∗∗*^
		NA	NA	NA	NA	NA	NA	NA
Deng *et al.* [20]	NA	NA	NA	NA	NA	NA	NA	NA
						3.22 (1.04–21.80)		65.00 (54.60, 78.75)^*∗∗∗∗*^
						109.25 (35.00–170.28)		89.00 (72.00, 133.50)^*∗∗∗∗*^
						NA		NA

Wang *et al.* [21]	1.19 (0.64, 2.56)	68 (43, 112)	0.07 (0.04, 0.18)	0.007 (0.006, 0.046)	1.02 (0.48, 3.69)	5 (5, 5)	NA	NA
	0.86 (0.57, 1.21)	57 (39, 88.5)	0.04 (0.03, 0.07)	0.044 (0.015, 0.131)	0.66 (0.31, 1.36)	5 (1.78, 5)		
	0.82 (0.56, 1.22)	97.5 (57.8, 236)	0.18 (0.1, 0.55)	0.044 (0.015, 0.131)	2.82 (0.91, 12.9)	5 (5, 5)		
	<0.001	<0.001	<0.001	<0.001	<0.001	<0.001		

Amit *et al.* [9]	NA	NA	NA	NA	NA	NA	NA	NA
					NA			
					NA			
					*p*=0.546			

Alamdari *et al.* [10]	NA	NA	NA	NA	NA	NA	NA	NA
					1.78 ± 2.14			1.68 ± 0.62
					3.39 ± 2.94			2.13 ± 0.75
					<0.0001			<0.0001

Stefano Ghio *et al.* [11]	NA	NA	NA	NA	NA	NA	NA	NA
				11 (5-25)				0.88 (0.72-1.1)
				31 (15-80)				1.09(0.88-1.61)
				<0.001				<0.001

Yukun *et al.* [22]	NA	NA	NA	15.3 (3.4, 37.7)^*∗∗*^	3.09(0.8,7.1)^*∗∗∗*^	NA	NA	80.0 (66.5, 99.8) ^*∗∗∗∗*^
				3.6 (0.3,18.9)^*∗∗*^	1.5(0.6, 3.1)^*∗∗∗*^			75(65.2, 83.6)^*∗∗∗∗*^
				31.4(11.0,84.2)^*∗∗*^	7.0(3.3,28.0)^*∗∗∗*^			93(74.2,125.3)^*∗∗∗∗*^
				<0.001	<0.001			0.017

NA= not applicable. ^*∗*^Measured in U/L, ^*∗∗*^measured in pg/ml, ^*∗∗∗*^measured in mg/L, and ^*∗∗∗∗*^ measured in *μ*mol/L.

**Table 5:** Cardiovascular complications.

	Shock	Acute cardiac injury/myocarditis	Heart failure	Arrhythmia
Author	Total	Total	Total	Total
	Survivor	Survivor	Survivor	Survivor
	Nonsurvivor	Nonsurvivor	Nonsurvivor	Nonsurvivor
	*p* value	*p* value	*p* value	*p* value

Wang *et al.* [12]	22 (20.6)	12 (11.2)	NA	NA
	3 (3.4)	4 (4.5)		
	19 (100.0)	8 (42.1)		
	NA	NA		

Yang *et al.* [13]	NA	NA	NA	NA

Zhou *et al.* [14]	38 (20%)	33 (17%)	44 (23%)	NA
	0	1 (1%)	16 (12%)	
	38 (70%)	32 (59%)	28 (52%)	
	<0·0001	<0·0001	<0·0001	

Si *et al.* [15]	NA	NA	NA	44
	NA	NA	NA	38
	23	NA	NA	6
	NA	NA	NA	NA

Shi *et al.* [16]	4 (6.5%)	20 (30.6%)	12 (19.4%)	NA
	NA	NA	NA	
	NA	NA	NA	
	NA	NA	NA	

Fang-fang Chen *et al*. [17]	NA	91	NA	NA
		NA		
		NA		
		NA		

Xie *et al.* [18]	147 (20%)	357 (59.2%)	NA	NA
	NA	NA		
	NA	NA		
	NA	NA		

Chen *et al*. [19]	46 (17)	89	43	NA
	0	17	2	
	46 (41)	72	41	
	NA	NA	NA	

Deng *et al.* [20]	13	66	NA	NA
	0	1 (0.9)		
	13 (11.9)	65 (59.6)		
	<0.001	<0.001		

Wang *et al.* [21]	56 (19.1%)	72 (24.6%)	NA	NA
	5 (2.8%)	5 (2.8%)		
	51 (44.0%)	67 (57.8%)		
	<0.001	<0.001		

Amit *et al.* [9]	29	8	11	NA
	9	3	2	
	20	5	9	
	0.113	0.694	0.079	

Alamdari *et al.* [10]	NA	NA	NA	53 [11.5%]
				23 [5.8%]
				30 [47.6%]
				<0.0001

Stefano Ghio *et al.* [12]	NA	NA	82	29
			51	12
			31	17
			0.25	0.002

Yukun *et al.* [22]	NA	NA	NA	NA

NA=Not applicable

## Data Availability

All the required information is available within the manuscript.

## Abbreviations

COVID-19: Coronavirus disease 19
SARS-CoV-2: Severe acute respiratory syndrome coronavirus 2
nCoV-2019: Novel coronavirus 2019
ACE: Angiotensin-converting enzyme.

## Supplementary Materials

Supplementary Materials NHLBI quality assessment tools for the observational cohort and cross-sectional studies.

## References

[1] Yonas E., Alwi I., Pranata R. (2020). Effect of heart failure on the outcome of COVID-19—A meta analysis and systematic review. *The American Journal of Emergency Medicine*.

[2] Lu R., Zhao X., Li J. (2020). Articles Genomic characterisation and epidemiology of 2019 novel coronavirus: implications for virus origins and receptor binding. *Lancet*.

[3] Adhikari S. P., Meng S., Wu Y. (2020). Epidemiology, causes, clinical manifestation and diagnosis, prevention and control of coronavirus disease (COVID-19) during the early outbreak period: a scoping review. *Infectious Diseases of Poverty*.

[4] Shafi A. M. A., Shaikh S. A., Shirke M. M., Iddawela S., Harky A. (2020). Cardiac manifestations in COVID—19 patients—a systematic review. *Journal of Cardiac Surgery*.

[5] Kochi A. N., Forleo G. B., Tondo C., Fassini G. M. (2020). Cardiac and arrhythmic complications in patients with COVID—19. *Journal of Cardiovascular Electrophysiology*.

[6] Ejaz H., Alsrhani A., Zafar A. (2020). COVID-19 and comorbidities: deleterious impact on infected patients. *Journal of Infection and Public Health*.

[7] Meng X., Deng Y., Dai Z., Meng Z. (2020). COVID-19 and anosmia: A review based on up-to-date knowledge. *American Journal of Otolaryngology*.

[8] Liberati A., Altman D. G., Tetzlaff J. (2009). The PRISMA statement for reporting systematic reviews and meta-analyses of studies that evaluate health care interventions: explanation and elaboration. *Journal of Clinical Epidemiology*.

[9] Amit M., Sorkin A., Chen J. (2020). Clinical course and outcomes of severe COVID-19: A national scale study. *Journal of Clinical Medicine*.

[10] Alamdari N. M., Afaghi S., Rahimi F. S. (2020). Mortality risk factors among hospitalized COVID-19 patients in a major referral center in Iran. *Tohoku Journal of Experimental Medicine*.

[11] Ghio S., Baldi E., Vicentini A. (2020). Cardiac involvement at presentation in patients hospitalized with COVID—19 and their outcome in a tertiary referral hospital in northern Italy. *Internal and Emergency Medicine*.

[12] Wang D., Yin Y., Hu C. (2020). Clinical course and outcome of 107 patients infected with the novel coronavirus, SARS-CoV-2, discharged from two hospitals in Wuhan, China. *Critical Care*.

[13] Li B., Yang J., Zhao F. (2020). Prevalence and impact of cardiovascular metabolic diseases on COVID—19 in China. *Clinical Research in Cardiology*.

[14] Zhou F., Yu T., Du R. (2020). Articles Clinical course and risk factors for mortality of adult inpatients with COVID-19 in Wuhan, China: a retrospective cohort study. *Lancet*.

[15] Si D., Du B., Ni L. (2020). Death, discharge and arrhythmias among patients with COVID-19 and cardiac injury. *Canadian Medical Association Journal*.

[16] Shi S., Qin M., Cai Y. (2020). Characteristics and Clinical Significance of Myocardial Injury in Patients with Severe Coronavirus Disease 2019. *European Heart Journal*.

[17] Chen F. f, Zhong M., Liu Y. (2020). The characteristics and outcomes of 681 severe cases with COVID-19 in China. *Journal of Critical Care*.

[18] Xie J., Wu W., Li S. (2020). Clinical characteristics and outcomes of critically ill patients with novel coronavirus infectious disease (COVID—19) in China: A Retrospective Multicenter Study. *Intensive Care Medicine*.

[19] Chen T., Wu D., Chen H. (2020). Clinical characteristics of 113 deceased patients with coronavirus disease 2019: retrospective study. *BMJ*.

[20] Deng Y., Liu W., Liu K. (2020). Clinical characteristics of fatal and recovered cases of coronavirus disease 2019 in Wuhan, China: a retrospective study. *Chinese Medical Journal*.

[21] Wang Z., Ye D., Wang M. (2020). Clinical features of COVID-19 patients with different outcomes in wuhan: A retrospective observational study. *BioMed Research International*.

[22] Cao Y., Wang A., Wei R., Zhang Y. (2020). An unexpected cause of pulmonary hypertension. *European heart journal*.

[23] Choi K. W., Chau T. N., Tsang O. (2003). Annals of internal medicine article outcomes and prognostic factors in 267 patients with severe acute respiratory syndrome in Hong Kong. *Annals of Internal Medicine*.

[24] Hong K. H., Choi J. P., Hong S. H. (2017). Predictors of mortality in middle east respiratory syndrome (MERS). *Thorax*.

[25] Conti P., Younes A. (2020). Coronavirus COV-19/sars-CoV-2 affects women less than men: clinical response to viral infection. *Journal of Biological Regulators & Homeostatic Agents*.

[26] Huang C., Wang Y., Li X. (2020). Articles Clinical features of patients infected with 2019 novel coronavirus in Wuhan, China. *Lancet*.

[27] Yang X., Yu Y., Xu J. (2020). Articles Clinical course and outcomes of critically ill patients with SARS-CoV-2 pneumonia in Wuhan, China: a single-centered, retrospective, observational study. *Lancet Respiratory Medicine*.

[28] Alraddadi B. M., Watson J. T., Almarashi A. (2014). Risk factors for primary Middle East respiratory syndrome coronavirus illness in humans, Saudi arabia, 2014. *Emerging Infectious Diseases*.

[29] Gupta R., Ghosh A., Kumar A., Misra A. (2020). Diabetes & metabolic syndrome: clinical research & reviews clinical considerations for patients with diabetes in times of COVID-19 epidemic. *Diabetes & Metabolic Syndrome*.

[30] Hassantabar S., Ahmadi M., Sharifi A. (2020). Diagnosis and detection of infected tissue of COVID-19 patients based on lung x-ray image using convolutional neural network approaches. *Chaos, Solitons & Fractals*.

[31] Davoudi A., Ahmadi M., Sharifi A. (2021). Studying the effect of taking statins before infection in the severity reduction of COVID-19 with machine learning. *BioMed Research International*.

[32] Abadi M. Q. H., Rahmati S., Sharifi A., Ahmadi M. (2021). HSSAGA: designation and scheduling of nurses for taking care of COVID-19 patients using novel method of Hybrid Salp Swarm Algorithm and Genetic Algorithm. *Applied Soft Computing*.

