# Supplementary material for: Cardiac Implications of COVID-19 in Deceased and Recovered Patients: A Systematic Review
Source: Interdiscip Perspect Infect Dis. 2022 Jun 15;2022:9119930. doi: 10.1155/2022/9119930 (PMC9204499; doi:10.1155/2022/9119930)
Supplement: Supplementary Materials — NHLBI quality assessment tools for the observational cohort and cross-sectional studies. [file 9119930.f1.doc]

**NHLBI quality assessment tool for observational cohort and cross-sectional studies**

**(Format Author: Scoring)**

Wang et. al^9^ Score:10/14

| Criteria | Yes | No | Other (CD, NR, NA) |
| --- | --- | --- | --- |
| 1. Was the research question or objective in this paper clearly stated? | **✓** |  |  |
| 2. Was the study population clearly specified and defined? | **✓** |  |  |
| 3. Was the participation rate of eligible persons at least 50%? | **✓** |  |  |
| 4. Were all the subjects selected or recruited from the same or similar populations (including the same time period)? Were inclusion and exclusion criteria for being in the study prespecified and applied uniformly to all participants? | **✓** |  |  |
| 5. Was a sample size justification, power description, or variance and effect estimates provided? |  | **✓** |  |
| 6. For the analyses in this paper, were the exposure(s) of interest measured prior to the outcome(s) being measured? | **✓** |  |  |
| 7. Was the timeframe sufficient so that one could reasonably expect to see an association between exposure and outcome if it existed? | **✓** |  |  |
| 8. For exposures that can vary in amount or level, did the study examine different levels of the exposure as related to the outcome (e.g., categories of exposure, or exposure measured as continuous variable)? |  | **✓** |  |
| 9. Were the exposure measures (independent variables) clearly defined, valid, reliable, and implemented consistently across all study participants? | **✓** |  |  |
| 10. Was the exposure(s) assessed more than once over time? |  | **✓** |  |
| 11. Were the outcome measures (dependent variables) clearly defined, valid, reliable, and implemented consistently across all study participants? | **✓** |  |  |
| 12. Were the outcome assessors blinded to the exposure status of participants? |  | **✓** |  |
| 13. Was loss to follow-up after baseline 20% or less? | **✓** |  |  |
| 14. Were key potential confounding variables measured and adjusted statistically for their impact on the relationship between exposure(s) and outcome(s)? | **✓** |  |  |

CD: cannot determine; NA: not applicable; NR: not reported

Yang et. al^16^ Score:11/14

| Criteria | Yes | No | Other (CD, NR, NA) |
| --- | --- | --- | --- |
| 1. Was the research question or objective in this paper clearly stated? | **✓** |  |  |
| 2. Was the study population clearly specified and defined? | **✓** |  |  |
| 3. Was the participation rate of eligible persons at least 50%? | **✓** |  |  |
| 4. Were all the subjects selected or recruited from the same or similar populations (including the same time period)? Were inclusion and exclusion criteria for being in the study prespecified and applied uniformly to all participants? | **✓** |  |  |
| 5. Was a sample size justification, power description, or variance and effect estimates provided? |  | **✓** |  |
| 6. For the analyses in this paper, were the exposure(s) of interest measured prior to the outcome(s) being measured? | **✓** |  |  |
| 7. Was the timeframe sufficient so that one could reasonably expect to see an association between exposure and outcome if it existed? | **✓** |  |  |
| 8. For exposures that can vary in amount or level, did the study examine different levels of the exposure as related to the outcome (e.g., categories of exposure, or exposure measured as continuous variable)? |  | **✓** |  |
| 9. Were the exposure measures (independent variables) clearly defined, valid, reliable, and implemented consistently across all study participants? | **✓** |  |  |
| 10. Was the exposure(s) assessed more than once over time? | **✓** |  |  |
| 11. Were the outcome measures (dependent variables) clearly defined, valid, reliable, and implemented consistently across all study participants? | **✓** |  |  |
| 12. Were the outcome assessors blinded to the exposure status of participants? |  | **✓** |  |
| 13. Was loss to follow-up after baseline 20% or less? | **✓** |  |  |
| 14. Were key potential confounding variables measured and adjusted statistically for their impact on the relationship between exposure(s) and outcome(s)? | **✓** |  |  |

CD: cannot determine; NA: not applicable; NR: not reported

Zhou et. al^10^ Score:10/14

| Criteria | Yes | No | Other (CD, NR, NA) |
| --- | --- | --- | --- |
| 1. Was the research question or objective in this paper clearly stated? | **✓** |  |  |
| 2. Was the study population clearly specified and defined? | **✓** |  |  |
| 3. Was the participation rate of eligible persons at least 50%? | **✓** |  |  |
| 4. Were all the subjects selected or recruited from the same or similar populations (including the same time period)? Were inclusion and exclusion criteria for being in the study prespecified and applied uniformly to all participants? | **✓** |  |  |
| 5. Was a sample size justification, power description, or variance and effect estimates provided? |  | **✓** |  |
| 6. For the analyses in this paper, were the exposure(s) of interest measured prior to the outcome(s) being measured? | **✓** |  |  |
| 7. Was the timeframe sufficient so that one could reasonably expect to see an association between exposure and outcome if it existed? | **✓** |  |  |
| 8. For exposures that can vary in amount or level, did the study examine different levels of the exposure as related to the outcome (e.g., categories of exposure, or exposure measured as continuous variable)? |  | **✓** |  |
| 9. Were the exposure measures (independent variables) clearly defined, valid, reliable, and implemented consistently across all study participants? | **✓** |  |  |
| 10. Was the exposure(s) assessed more than once over time? |  | **✓** |  |
| 11. Were the outcome measures (dependent variables) clearly defined, valid, reliable, and implemented consistently across all study participants? | **✓** |  |  |
| 12. Were the outcome assessors blinded to the exposure status of participants? |  | **✓** |  |
| 13. Was loss to follow-up after baseline 20% or less? | **✓** |  |  |
| 14. Were key potential confounding variables measured and adjusted statistically for their impact on the relationship between exposure(s) and outcome(s)? | **✓** |  |  |

CD: cannot determine; NA: not applicable; NR: not reported

Si et. al^21^ Score:10/14

| Criteria | Yes | No | Other (CD, NR, NA) |
| --- | --- | --- | --- |
| 1. Was the research question or objective in this paper clearly stated? | **✓** |  |  |
| 2. Was the study population clearly specified and defined? | **✓** |  |  |
| 3. Was the participation rate of eligible persons at least 50%? | **✓** |  |  |
| 4. Were all the subjects selected or recruited from the same or similar populations (including the same time period)? Were inclusion and exclusion criteria for being in the study prespecified and applied uniformly to all participants? | **✓** |  |  |
| 5. Was a sample size justification, power description, or variance and effect estimates provided? |  | **✓** |  |
| 6. For the analyses in this paper, were the exposure(s) of interest measured prior to the outcome(s) being measured? | **✓** |  |  |
| 7. Was the timeframe sufficient so that one could reasonably expect to see an association between exposure and outcome if it existed? | **✓** |  |  |
| 8. For exposures that can vary in amount or level, did the study examine different levels of the exposure as related to the outcome (e.g., categories of exposure, or exposure measured as continuous variable)? |  | **✓** |  |
| 9. Were the exposure measures (independent variables) clearly defined, valid, reliable, and implemented consistently across all study participants? | **✓** |  |  |
| 10. Was the exposure(s) assessed more than once over time? |  | **✓** |  |
| 11. Were the outcome measures (dependent variables) clearly defined, valid, reliable, and implemented consistently across all study participants? | **✓** |  |  |
| 12. Were the outcome assessors blinded to the exposure status of participants? |  | **✓** |  |
| 13. Was loss to follow-up after baseline 20% or less? | **✓** |  |  |
| 14. Were key potential confounding variables measured and adjusted statistically for their impact on the relationship between exposure(s) and outcome(s)? | **✓** |  |  |

CD: cannot determine; NA: not applicable; NR: not reported

Shi et. al^17^ Score:9/13

| Criteria | Yes | No | Other (CD, NR, NA) |
| --- | --- | --- | --- |
| 1. Was the research question or objective in this paper clearly stated? | **✓** |  |  |
| 2. Was the study population clearly specified and defined? | **✓** |  |  |
| 3. Was the participation rate of eligible persons at least 50%? | **✓** |  |  |
| 4. Were all the subjects selected or recruited from the same or similar populations (including the same time period)? Were inclusion and exclusion criteria for being in the study prespecified and applied uniformly to all participants? | **✓** |  |  |
| 5. Was a sample size justification, power description, or variance and effect estimates provided? |  | **✓** |  |
| 6. For the analyses in this paper, were the exposure(s) of interest measured prior to the outcome(s) being measured? | **✓** |  |  |
| 7. Was the timeframe sufficient so that one could reasonably expect to see an association between exposure and outcome if it existed? |  | **✓** |  |
| 8. For exposures that can vary in amount or level, did the study examine different levels of the exposure as related to the outcome (e.g., categories of exposure, or exposure measured as continuous variable)? |  | **✓** |  |
| 9. Were the exposure measures (independent variables) clearly defined, valid, reliable, and implemented consistently across all study participants? | **✓** |  |  |
| 10. Was the exposure(s) assessed more than once over time? |  | **✓** |  |
| 11. Were the outcome measures (dependent variables) clearly defined, valid, reliable, and implemented consistently across all study participants? | **✓** |  |  |
| 12. Were the outcome assessors blinded to the exposure status of participants? |  | **✓** |  |
| 13. Was loss to follow-up after baseline 20% or less? |  |  | CD |
| 14. Were key potential confounding variables measured and adjusted statistically for their impact on the relationship between exposure(s) and outcome(s)? | **✓** |  |  |

CD: cannot determine; NA: not applicable; NR: not reported

Fang-fang Chen et. al^13^ Score:10/14

| Criteria | Yes | No | Other (CD, NR, NA) |
| --- | --- | --- | --- |
| 1. Was the research question or objective in this paper clearly stated? | **✓** |  |  |
| 2. Was the study population clearly specified and defined? | **✓** |  |  |
| 3. Was the participation rate of eligible persons at least 50%? | **✓** |  |  |
| 4. Were all the subjects selected or recruited from the same or similar populations (including the same time period)? Were inclusion and exclusion criteria for being in the study prespecified and applied uniformly to all participants? | **✓** |  |  |
| 5. Was a sample size justification, power description, or variance and effect estimates provided? |  | **✓** |  |
| 6. For the analyses in this paper, were the exposure(s) of interest measured prior to the outcome(s) being measured? | **✓** |  |  |
| 7. Was the timeframe sufficient so that one could reasonably expect to see an association between exposure and outcome if it existed? | **✓** |  |  |
| 8. For exposures that can vary in amount or level, did the study examine different levels of the exposure as related to the outcome (e.g., categories of exposure, or exposure measured as continuous variable)? |  | **✓** |  |
| 9. Were the exposure measures (independent variables) clearly defined, valid, reliable, and implemented consistently across all study participants? | **✓** |  |  |
| 10. Was the exposure(s) assessed more than once over time? |  | **✓** |  |
| 11. Were the outcome measures (dependent variables) clearly defined, valid, reliable, and implemented consistently across all study participants? | **✓** |  |  |
| 12. Were the outcome assessors blinded to the exposure status of participants? |  | **✓** |  |
| 13. Was loss to follow-up after baseline 20% or less? | **✓** |  |  |
| 14. Were key potential confounding variables measured and adjusted statistically for their impact on the relationship between exposure(s) and outcome(s)? | **✓** |  |  |

CD: cannot determine; NA: not applicable; NR: not reported

Xie et. al^12^ Score:10/14

| Criteria | Yes | No | Other (CD, NR, NA) |
| --- | --- | --- | --- |
| 1. Was the research question or objective in this paper clearly stated? | **✓** |  |  |
| 2. Was the study population clearly specified and defined? | **✓** |  |  |
| 3. Was the participation rate of eligible persons at least 50%? | **✓** |  |  |
| 4. Were all the subjects selected or recruited from the same or similar populations (including the same time period)? Were inclusion and exclusion criteria for being in the study prespecified and applied uniformly to all participants? | **✓** |  |  |
| 5. Was a sample size justification, power description, or variance and effect estimates provided? |  | **✓** |  |
| 6. For the analyses in this paper, were the exposure(s) of interest measured prior to the outcome(s) being measured? | **✓** |  |  |
| 7. Was the timeframe sufficient so that one could reasonably expect to see an association between exposure and outcome if it existed? | **✓** |  |  |
| 8. For exposures that can vary in amount or level, did the study examine different levels of the exposure as related to the outcome (e.g., categories of exposure, or exposure measured as continuous variable)? |  | **✓** |  |
| 9. Were the exposure measures (independent variables) clearly defined, valid, reliable, and implemented consistently across all study participants? | **✓** |  |  |
| 10. Was the exposure(s) assessed more than once over time? |  | **✓** |  |
| 11. Were the outcome measures (dependent variables) clearly defined, valid, reliable, and implemented consistently across all study participants? | **✓** |  |  |
| 12. Were the outcome assessors blinded to the exposure status of participants? |  | **✓** |  |
| 13. Was loss to follow-up after baseline 20% or less? | **✓** |  |  |
| 14. Were key potential confounding variables measured and adjusted statistically for their impact on the relationship between exposure(s) and outcome(s)? | **✓** |  |  |

CD: cannot determine; NA: not applicable; NR: not reported

Chen et. al^19^ Score:10/14

| Criteria | Yes | No | Other (CD, NR, NA) |
| --- | --- | --- | --- |
| 1. Was the research question or objective in this paper clearly stated? | **✓** |  |  |
| 2. Was the study population clearly specified and defined? | **✓** |  |  |
| 3. Was the participation rate of eligible persons at least 50%? | **✓** |  |  |
| 4. Were all the subjects selected or recruited from the same or similar populations (including the same time period)? Were inclusion and exclusion criteria for being in the study prespecified and applied uniformly to all participants? | **✓** |  |  |
| 5. Was a sample size justification, power description, or variance and effect estimates provided? |  | **✓** |  |
| 6. For the analyses in this paper, were the exposure(s) of interest measured prior to the outcome(s) being measured? | **✓** |  |  |
| 7. Was the timeframe sufficient so that one could reasonably expect to see an association between exposure and outcome if it existed? | **✓** |  |  |
| 8. For exposures that can vary in amount or level, did the study examine different levels of the exposure as related to the outcome (e.g., categories of exposure, or exposure measured as continuous variable)? |  | **✓** |  |
| 9. Were the exposure measures (independent variables) clearly defined, valid, reliable, and implemented consistently across all study participants? | **✓** |  |  |
| 10. Was the exposure(s) assessed more than once over time? |  | **✓** |  |
| 11. Were the outcome measures (dependent variables) clearly defined, valid, reliable, and implemented consistently across all study participants? | **✓** |  |  |
| 12. Were the outcome assessors blinded to the exposure status of participants? |  | **✓** |  |
| 13. Was loss to follow-up after baseline 20% or less? | **✓** |  |  |
| 14. Were key potential confounding variables measured and adjusted statistically for their impact on the relationship between exposure(s) and outcome(s)? | **✓** |  |  |

CD: cannot determine; NA: not applicable; NR: not reported

Deng et. al^18^ Score:11/14

| Criteria | Yes | No | Other (CD, NR, NA) |
| --- | --- | --- | --- |
| 1. Was the research question or objective in this paper clearly stated? | **✓** |  |  |
| 2. Was the study population clearly specified and defined? | **✓** |  |  |
| 3. Was the participation rate of eligible persons at least 50%? | **✓** |  |  |
| 4. Were all the subjects selected or recruited from the same or similar populations (including the same time period)? Were inclusion and exclusion criteria for being in the study prespecified and applied uniformly to all participants? | **✓** |  |  |
| 5. Was a sample size justification, power description, or variance and effect estimates provided? |  | **✓** |  |
| 6. For the analyses in this paper, were the exposure(s) of interest measured prior to the outcome(s) being measured? | **✓** |  |  |
| 7. Was the timeframe sufficient so that one could reasonably expect to see an association between exposure and outcome if it existed? | **✓** |  |  |
| 8. For exposures that can vary in amount or level, did the study examine different levels of the exposure as related to the outcome (e.g., categories of exposure, or exposure measured as continuous variable)? | **✓** |  |  |
| 9. Were the exposure measures (independent variables) clearly defined, valid, reliable, and implemented consistently across all study participants? | **✓** |  |  |
| 10. Was the exposure(s) assessed more than once over time? |  | **✓** |  |
| 11. Were the outcome measures (dependent variables) clearly defined, valid, reliable, and implemented consistently across all study participants? | **✓** |  |  |
| 12. Were the outcome assessors blinded to the exposure status of participants? |  | **✓** |  |
| 13. Was loss to follow-up after baseline 20% or less? | **✓** |  |  |
| 14. Were key potential confounding variables measured and adjusted statistically for their impact on the relationship between exposure(s) and outcome(s)? | **✓** |  |  |

CD: cannot determine; NA: not applicable; NR: not reported

Wang et. al^20^ Score:10/14

| Criteria | Yes | No | Other (CD, NR, NA) |
| --- | --- | --- | --- |
| 1. Was the research question or objective in this paper clearly stated? | **✓** |  |  |
| 2. Was the study population clearly specified and defined? | **✓** |  |  |
| 3. Was the participation rate of eligible persons at least 50%? | **✓** |  |  |
| 4. Were all the subjects selected or recruited from the same or similar populations (including the same time period)? Were inclusion and exclusion criteria for being in the study prespecified and applied uniformly to all participants? | **✓** |  |  |
| 5. Was a sample size justification, power description, or variance and effect estimates provided? |  | **✓** |  |
| 6. For the analyses in this paper, were the exposure(s) of interest measured prior to the outcome(s) being measured? | **✓** |  |  |
| 7. Was the timeframe sufficient so that one could reasonably expect to see an association between exposure and outcome if it existed? | **✓** |  |  |
| 8. For exposures that can vary in amount or level, did the study examine different levels of the exposure as related to the outcome (e.g., categories of exposure, or exposure measured as continuous variable)? |  | **✓** |  |
| 9. Were the exposure measures (independent variables) clearly defined, valid, reliable, and implemented consistently across all study participants? | **✓** |  |  |
| 10. Was the exposure(s) assessed more than once over time? |  | **✓** |  |
| 11. Were the outcome measures (dependent variables) clearly defined, valid, reliable, and implemented consistently across all study participants? | **✓** |  |  |
| 12. Were the outcome assessors blinded to the exposure status of participants? |  | **✓** |  |
| 13. Was loss to follow-up after baseline 20% or less? | **✓** |  |  |
| 14. Were key potential confounding variables measured and adjusted statistically for their impact on the relationship between exposure(s) and outcome(s)? | **✓** |  |  |

CD: cannot determine; NA: not applicable; NR: not reported

Amit et. al^11^ Score:11/14

| Criteria | Yes | No | Other (CD, NR, NA) |
| --- | --- | --- | --- |
| 1. Was the research question or objective in this paper clearly stated? | **✓** |  |  |
| 2. Was the study population clearly specified and defined? | **✓** |  |  |
| 3. Was the participation rate of eligible persons at least 50%? | **✓** |  |  |
| 4. Were all the subjects selected or recruited from the same or similar populations (including the same time period)? Were inclusion and exclusion criteria for being in the study prespecified and applied uniformly to all participants? | **✓** |  |  |
| 5. Was a sample size justification, power description, or variance and effect estimates provided? |  | **✓** |  |
| 6. For the analyses in this paper, were the exposure(s) of interest measured prior to the outcome(s) being measured? | **✓** |  |  |
| 7. Was the timeframe sufficient so that one could reasonably expect to see an association between exposure and outcome if it existed? | **✓** |  |  |
| 8. For exposures that can vary in amount or level, did the study examine different levels of the exposure as related to the outcome (e.g., categories of exposure, or exposure measured as continuous variable)? |  | **✓** |  |
| 9. Were the exposure measures (independent variables) clearly defined, valid, reliable, and implemented consistently across all study participants? | **✓** |  |  |
| 10. Was the exposure(s) assessed more than once over time? | **✓** |  |  |
| 11. Were the outcome measures (dependent variables) clearly defined, valid, reliable, and implemented consistently across all study participants? | **✓** |  |  |
| 12. Were the outcome assessors blinded to the exposure status of participants? |  | **✓** |  |
| 13. Was loss to follow-up after baseline 20% or less? | **✓** |  |  |
| 14. Were key potential confounding variables measured and adjusted statistically for their impact on the relationship between exposure(s) and outcome(s)? | **✓** |  |  |

CD: cannot determine; NA: not applicable; NR: not reported

Alamdari et. al^14^ Score:10/14

| Criteria | Yes | No | Other (CD, NR, NA) |
| --- | --- | --- | --- |
| 1. Was the research question or objective in this paper clearly stated? | **✓** |  |  |
| 2. Was the study population clearly specified and defined? | **✓** |  |  |
| 3. Was the participation rate of eligible persons at least 50%? | **✓** |  |  |
| 4. Were all the subjects selected or recruited from the same or similar populations (including the same time period)? Were inclusion and exclusion criteria for being in the study prespecified and applied uniformly to all participants? | **✓** |  |  |
| 5. Was a sample size justification, power description, or variance and effect estimates provided? |  | **✓** |  |
| 6. For the analyses in this paper, were the exposure(s) of interest measured prior to the outcome(s) being measured? | **✓** |  |  |
| 7. Was the timeframe sufficient so that one could reasonably expect to see an association between exposure and outcome if it existed? | **✓** |  |  |
| 8. For exposures that can vary in amount or level, did the study examine different levels of the exposure as related to the outcome (e.g., categories of exposure, or exposure measured as continuous variable)? |  | **✓** |  |
| 9. Were the exposure measures (independent variables) clearly defined, valid, reliable, and implemented consistently across all study participants? | **✓** |  |  |
| 10. Was the exposure(s) assessed more than once over time? |  | **✓** |  |
| 11. Were the outcome measures (dependent variables) clearly defined, valid, reliable, and implemented consistently across all study participants? | **✓** |  |  |
| 12. Were the outcome assessors blinded to the exposure status of participants? |  | **✓** |  |
| 13. Was loss to follow-up after baseline 20% or less? | **✓** |  |  |
| 14. Were key potential confounding variables measured and adjusted statistically for their impact on the relationship between exposure(s) and outcome(s)? | **✓** |  |  |

CD: cannot determine; NA: not applicable; NR: not reported

Stephano Ghio et. al^15^ Score:9/14

| Criteria | Yes | No | Other (CD, NR, NA) |
| --- | --- | --- | --- |
| 1. Was the research question or objective in this paper clearly stated? | **✓** |  |  |
| 2. Was the study population clearly specified and defined? | **✓** |  |  |
| 3. Was the participation rate of eligible persons at least 50%? | **✓** |  |  |
| 4. Were all the subjects selected or recruited from the same or similar populations (including the same time period)? Were inclusion and exclusion criteria for being in the study prespecified and applied uniformly to all participants? |  | **✓** |  |
| 5. Was a sample size justification, power description, or variance and effect estimates provided? |  | **✓** |  |
| 6. For the analyses in this paper, were the exposure(s) of interest measured prior to the outcome(s) being measured? | **✓** |  |  |
| 7. Was the timeframe sufficient so that one could reasonably expect to see an association between exposure and outcome if it existed? | **✓** |  |  |
| 8. For exposures that can vary in amount or level, did the study examine different levels of the exposure as related to the outcome (e.g., categories of exposure, or exposure measured as continuous variable)? |  | **✓** |  |
| 9. Were the exposure measures (independent variables) clearly defined, valid, reliable, and implemented consistently across all study participants? | **✓** |  |  |
| 10. Was the exposure(s) assessed more than once over time? |  | **✓** |  |
| 11. Were the outcome measures (dependent variables) clearly defined, valid, reliable, and implemented consistently across all study participants? | **✓** |  |  |
| 12. Were the outcome assessors blinded to the exposure status of participants? |  | **✓** |  |
| 13. Was loss to follow-up after baseline 20% or less? | **✓** |  |  |
| 14. Were key potential confounding variables measured and adjusted statistically for their impact on the relationship between exposure(s) and outcome(s)? | **✓** |  |  |

CD: cannot determine; NA: not applicable; NR: not reported

Yukun et. al^22^ Score:10/14

| Criteria | Yes | No | Other (CD, NR, NA) |
| --- | --- | --- | --- |
| 1. Was the research question or objective in this paper clearly stated? | **✓** |  |  |
| 2. Was the study population clearly specified and defined? | **✓** |  |  |
| 3. Was the participation rate of eligible persons at least 50%? | **✓** |  |  |
| 4. Were all the subjects selected or recruited from the same or similar populations (including the same time period)? Were inclusion and exclusion criteria for being in the study prespecified and applied uniformly to all participants? | **✓** |  |  |
| 5. Was a sample size justification, power description, or variance and effect estimates provided? |  | **✓** |  |
| 6. For the analyses in this paper, were the exposure(s) of interest measured prior to the outcome(s) being measured? | **✓** |  |  |
| 7. Was the timeframe sufficient so that one could reasonably expect to see an association between exposure and outcome if it existed? | **✓** |  |  |
| 8. For exposures that can vary in amount or level, did the study examine different levels of the exposure as related to the outcome (e.g., categories of exposure, or exposure measured as continuous variable)? |  | **✓** |  |
| 9. Were the exposure measures (independent variables) clearly defined, valid, reliable, and implemented consistently across all study participants? | **✓** |  |  |
| 10. Was the exposure(s) assessed more than once over time? |  | **✓** |  |
| 11. Were the outcome measures (dependent variables) clearly defined, valid, reliable, and implemented consistently across all study participants? | **✓** |  |  |
| 12. Were the outcome assessors blinded to the exposure status of participants? |  | **✓** |  |
| 13. Was loss to follow-up after baseline 20% or less? | **✓** |  |  |
| 14. Were key potential confounding variables measured and adjusted statistically for their impact on the relationship between exposure(s) and outcome(s)? | **✓** |  |  |

CD: cannot determine; NA: not applicable; NR: not reported
